# Evaluation of the diabetes care cascade and compliance with WHO global coverage targets in Iran based on STEPS survey 2021

**DOI:** 10.1038/s41598-023-39433-7

**Published:** 2023-08-19

**Authors:** Sina Azadnajafabad, Naser Ahmadi, Negar Rezaei, Mohammad-Mahdi Rashidi, Sahar Saeedi Moghaddam, Esmaeil Mohammadi, Mohsen Abbasi-Kangevari, Mohammadreza Naderian, Erfan Ghasemi, Yosef Farzi, Ameneh Kazemi, Arezou Dilmaghani-Marand, Moein Yoosefi, Shahabeddin Rezaei, Maryam Nasserinejad, Nima Fattahi, Nazila Rezaei, Rosa Haghshenas, Elmira Foroutan Mehr, Sogol Koolaji, Farideh Razi, Shirin Djalalinia, Bagher Larijani, Farshad Farzadfar

**Affiliations:** 1 Non-Communicable Diseases Research Center, Endocrinology and Metabolism Population Sciences Institute, Tehran University of Medical Sciences, Tehran, Iran; 2 Kiel Institute for the World Economy, Kiel, Germany; 3 Department of Neurological Surgery, University of Oklahoma Health Sciences Center, Oklahoma City, OK USA; 4 Tehran Heart Center, Cardiovascular Diseases Research Institute, Tehran University of Medical Sciences, Tehran, Iran; 5 Department of Mathematics and Statistics, Memorial University of Newfoundland, St. John’s, NL Canada; 6 Human Nutrition Program, Department of Human Sciences, The Ohio State University, Columbus, OH USA; 7 Faculty of Medicine, Center for Life Course Health Research, University of Oulu, Oulu, Finland; 8 Department of Internal Medicine, Yale School of Medicine, New Haven, CT USA; 9 Metabolomics and Genomics Research Center, Endocrinology and Metabolism Research Institute, Tehran University of Medical Sciences, Tehran, Iran; 10 Development of Research and Technology Center, Deputy of Research and Technology, Ministry of Health and Medical Education, Tehran, Iran; 11 Endocrinology and Metabolism Research Center, Endocrinology and Metabolism Clinical Sciences Institute, Tehran University of Medical Sciences, Tehran, Iran; 12 Non-Communicable Diseases Research Center, Endocrinology and Metabolism Population Sciences Institute, Tehran University of Medical Sciences, Second Floor, No.10, Jalal Al-E-Ahmad Highway, Tehran, 1411713137 Iran

**Keywords:** Diseases, Endocrinology, Health care, Medical research

## Abstract

This study aimed to investigate the diabetes mellitus (DM) and prediabetes epidemiology, care cascade, and compliance with global coverage targets. We recruited the results of the nationally representative Iran STEPS Survey 2021. Diabetes and prediabetes were two main outcomes. Diabetes awareness, treatment coverage, and glycemic control were calculated for all population with diabetes to investigate the care cascade. Four global coverage targets for diabetes developed by the World Health Organization were adopted to assess the DM diagnosis and control status. Among 18,119 participants, the national prevalence of DM and prediabetes were 14.2% (95% confidence interval 13.4–14.9) and 24.8% (23.9–25.7), respectively. The prevalence of DM treatment coverage was 65.0% (62.4–67.7), while the prevalence of good (HbA1C < 7%) glycemic control was 28.0% (25.0–31.0) among all individuals with diabetes. DM diagnosis and statin use statics were close to global targets (73.3% vs 80%, and 50.1% vs 60%); however, good glycemic control and strict blood pressure control statistics, were much way behind the goals (36.7% vs 80%, and 28.5% vs 80%). A major proportion of the Iranian population are affected by DM and prediabetes, and glycemic control is poorly achieved, indicating a sub-optimal care for diabetes and comorbidities like hypertension.

## Introduction

Diabetes mellitus (DM) is one of the major burdensome noncommunicable diseases (NCDs) and is responsible for a significant share of premature mortality due to NCDs^1^. During the past decades, DM and its complications’ prevalence and burden have increased, afflicting almost all countries and regions with different socioeconomic states^2^. Recent projections on the prevalence and burden of DM for the upcoming decade also have proved the continuing rising trends, with worrying patterns in countries with lower-income^1^. The economic burden of DM is another significant burden shown to be considerable in both rich and poor regions of world, and predictions show a growth in DM economic burden in future, even-though some achievements in diabetes control goals^3^.

The care cascade which was first developed to examine the care continua for communicable diseases^4^, indicates the cascade of disease diagnosis, treatment, and control which also had promising results on the evaluation of provided care for NCDs like diabetes^5^. Pooled analysis of nationally representative surveys on diabetes care cascade in low- and middle-income countries (LMICs) have reported poor diabetes management in these countries and there is a huge unmet need in all stage of DM diagnosis, treatment, control in these areas^6^. In order to set targets for achieving diabetes control globally, the World Health Organization (WHO) came up with the first-ever global coverage targets for diabetes decided at the 75th World Health Assembly held on May 2022 and proposed five targets for DM coverage by 2030, including targets on diabetes diagnosis, glycemic control, blood pressure control, statin use, and a goal specified for type 1 DM^7^.

Iran as a developing country with its transitioning status to the epidemic of NCDs is majorly encountered with the burden of DM^8^. This country includes geographically, ethnically, and socioeconomically diverse regions and populations, and estimations on the prevalence of DM have led to different statistics; however, national surveys showed an estimated prevalence of DM about 10–15% and prediabetes about 25–31%^9,10^. Also, diabetes awareness, treatment, and control differ between studies as investigated samples and methods vary; therefore, reaching exact estimations is challenging^9,11^. Based on recent nationally representative surveys of Iranian adults, about 80% of patients with diabetes were aware of their condition, but glycemic control was achieved in about 41% of whom received treatment^9^. Also, according to the previous round of a similar national survey of NCD risk factors, barely more than half (52%) of the patients with self-reported DM were under strict glycemic control^10^. It is well-known and investigated that improving DM awareness and treatment lead to better control of the disease^12^; therefore, focused plans should be planned to facilitate the surveillance and improvement of these factors^13^. High fasting plasma glucose (FPG) as a precursor responsible for prediabetes and DM is one of the leading NCDs risk factors in Iran, which also contributes to many other chronic conditions like cardiovascular diseases^14^.

WHO has proposed the STEPwise Approach to NCD Risk Factor Surveillance (STEPS) framework as a standard measure for surveillance of NCDs risk factors^15^. Iran’s health system could successfully run the latest Iran STEPS Survey 2021 during the COVID-19 pandemic^16^. Here, we present the results of survey on the prevalence of DM and prediabetes and chief aspects of diabetes care cascade and based on the recent WHO global coverage targets.

## Methods

### Study design

Comprehensive details of the Iran STEPS Survey 2021 are provided elsewhere in a study protocol^16^. This survey had two main phases of design and implementation including three steps of data collection via questionnaires, physical measurements, and laboratory assessments. The first step of the survey was designed based on the latest version of the WHO STEPS instrument, version 3.2^17^. The second step of measured participants’ weight, height, hip circumference, waist circumference, pulse rate, and blood pressure according to defined standards in the survey protocol. The third step of laboratory measurements happened at the survey headquarter using the auto-analyzer (Roche-Hitachi Cobas C311, High–Technologies Corporation, Tokyo, Japan) approved by the reference laboratory^16^.

### Study population

To make this survey nationally representative, a clustered sampling method was used to recruit samples among Iranian adults aged ≥ 18 years old from urban and rural areas of all 31 provinces of Iran. A total number of 28,821 individuals were calculated for inclusion in survey, which whom 27,874 completed the first step, 27,745 completed the second step, and 18,119 completed the third step. The current study on the prevalence of DM, prediabetes, and related factors were done only on the population aged ≥ 25 years old.

### Definition of variables

Different variables in the collected dataset of survey were used to estimate the prevalence of DM and prediabetes as the primary outcomes. FPG and whole blood Hemoglobin A1c (HbA1c) were the key used laboratory tests. Diabetes was defined as FPG ≥ 126 mg/dL (7.0 mmol/L) *or* taking oral antihyperglycemic drugs/insulin based on self-reports. This study made no distinction between type 1 and 2 DM. Prediabetes was defined as 100 < FPG ≤ 125 mg/dL (5.6–6.9 mmol/L) based on laboratory measurement, excluding those having diabetes with mentioned criteria. DM awareness was assessed based on self-report, asking “Has any healthcare worker told you that your blood sugar is high or you have diabetes?” among all defined with diabetes. DM treatment coverage was assessed based on self-report asking “Do you currently use any oral antihyperglycemic agent or insulin for hyperglycemia or diabetes?” among all with diabetes. DM control was defined as good glycemic control in HbA1c < 7% and fair glycemic control in HbA1c < 8%, among all with diabetes^18^. Categories of antihyperglycemic agents were classified into (A) non-insulin drugs, (B) insulins, and (C) herbal medicine. Experiencing hypoglycemia in the past two weeks was assessed by recording patients' self-reported signs and symptoms of hypoglycemia.

### WHO global coverage targets

Based on the WHO global coverage targets for diabetes^7^, we adopted the first four targets of (1) 80% diagnosis of diabetes, (2) 80% good glycemic control in diagnosed cases, (3) 80% good blood pressure control in diagnosed cases, and (4) 60% receiving statins in patients aged ≥ 40 years, and excluded the fifth target as we did not distinct types of DM in this survey. Target 1 was equivalent to DM awareness, target 2 was equivalent to glycemic control criteria among those being aware of the condition, target 3 was calculated bases on two cut-off of systolic/diastolic blood pressure of < 140/90 and < 130/80^19^, and target 4 was calculated according to the use of statins for primary or secondary prevention among patients with diabetes.

### Other study variables

Wealth index (WI) as the implemented socioeconomic stratification for the population included in this survey, was calculated using the collected data on the household assets via questionnaires, and the values were categorized into five quintiles of poorest (first quintile) to wealthiest (fifth quintile)^16^. Years of schooling was the measure of assessed education reported in four categories [0, 1–6, 7–11, and ≥ 12]. Insurance status was assessed by asking about basic and complementary insurance coverage. Health-related quality of life (HRQoL) was assessed by the EuroQol five-dimensional at three levels (EQ-5D-3L) questionnaire evaluating mobility, self-care, usual activities, pain/discomfort, and anxiety/depression, which was previously validated for the Iranian population^16^. The participants’ residency was stratified into rural or urban areas. Age of the participants was reported in six categories [25–34, 35–44, 45–54, 55–64, 65–74, and ≥ 75].

### Statistical analysis

After survey data collection, data cleaning and weighting process were conducted by two expert biostatisticians. Age, sex, and area of residency standardizations were made based on the standard Iran population data extracted from the Iran census 2016 as the most recently available data^16^. Estimations of prevalence (in percentage per population) were made in addition to the 95% confidence interval (95% CI). The significant difference between various interest groups was defined if the estimated 95% CI of outcomes did not cross. Data cleaning and analysis were done using STATA version 14 (STATA Corp., College Station, Texas, USA) and R statistical package version 4.1.2 (https://cran.r-project.org).

### Ethical considerations

All participants of the STEPS survey were informed about the methods and goals of the survey and their participation was voluntary after providing written informed consent. The ethical committee of the National Institute for Health Research reviewed and approved the survey protocol (ID: IR.TUMS.NIHR.REC.1398.006). The current investigation was designed and performed in accordance with relevant instituitional guidelines/regulations and the Declaration of Helsinki.

## Results

### Prevalence of diabetes and prediabetes

**Figure 1:**
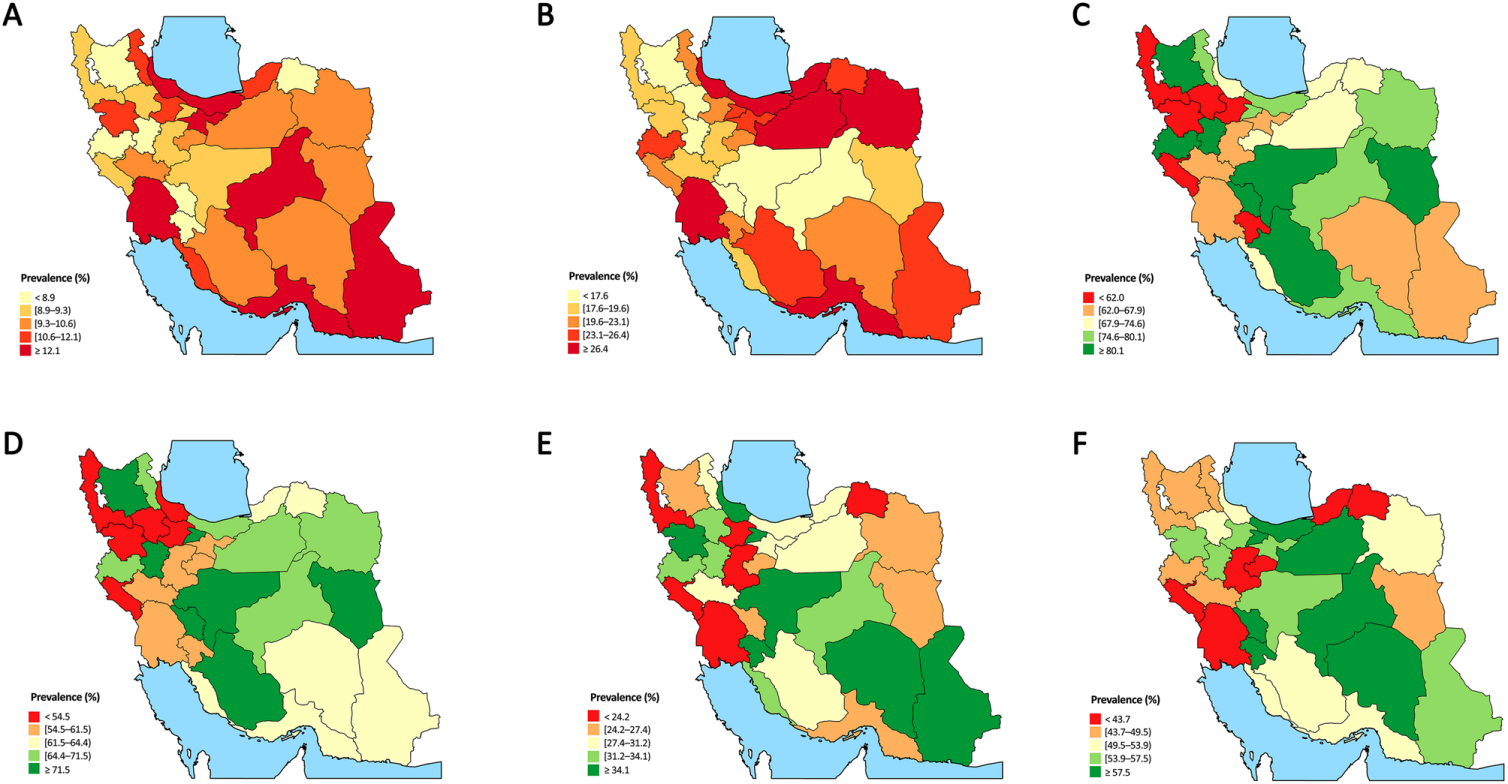
Subnational map of prevalence of (**A**) diabetes, (**B**) prediabetes, (**C**) diabetes awareness, (**D**) diabetes treatment coverage, (**E**) good glycemic control (HbA1C < 7%), and (**F**) fair glycemic control (HbA1C < 8%) in Iran STEPS Survey 2021 [Maps in this figure are originally generated using the Iran STEPS Survey 2021 data and by R programming language v4.1.2 (https://cran.r-project.org/)].

The total prevalence of diabetes at the national level was 14.2% (95% CI 13.4–14.9) for both sexes, 13.5% (12.3–14.6) for males, and 14.7% (13.8–15.7) for females. There was an increasing pattern of prevalence of diabetes with the aging population. People who resided in urban areas had a significantly higher prevalence of diabetes (15.2% [14.3–16.1]) compared to rural population (11.0% [10.0–12.0]). The prevalence of diabetes was noticeably higher in the population with the least education and vice versa (21.4% [19.6–23.2] vs 10.0% [8.8–11.2]). People with basic and complementary insurance showed to have significantly higher diabetes prevalence compared to populations without these health benefits (Table 1). Among provinces, prevalence of diabetes was lowest in Kermanshah (5.8% [3.6–8.1]) and highest in Khuzestan (16.3% [13.4–19.2]) (Fig. 1A).

**Table 1:** Prevalence of diabetes, prediabetes, diabetes awareness, treatment coverage, and glycemic cintrol for both sexes by population characteristics in Iran STEPS Survey 2021.

Categories	Subcategories	Prevalence% (95% confidence interval)					
		Diabetes	Prediabetes	Diabetes awareness	Diabetes treatment coverage	Good glycemic control	Fair glycemic control
Age	34–25	2.1 (1.5–2.7)	15.7 (14.0–17.4)	38.8 (24.4–53.2)	25.0 (13.0–37.0)	48.6 (22.2–75.0)	61.4 (35.8–87.0)
	44–35	5.7 (4.7–6.8)	23.2 (21.4–25.0)	64.5 (55.3–73.6)	53.7 (44.2–63.2)	29.4 (19.1–39.7)	42.1 (29.5–54.8)
	54–45	15.9 (14.3–17.5)	27.6 (25.7–29.5)	70.0 (64.6–75.4)	62.3 (56.8–67.8)	27.4 (21.0–33.7)	50.4 (43.3–57.6)
	64–55	26.8 (24.7–29.0)	28.8 (26.5–31.0)	78.4 (74.6–82.2)	70.4 (66.0–74.8)	27.6 (22.7–32.5)	51.9 (46.1–57.7)
	74–65	27.0 (24.4–29.7)	30.0 (26.9–33.1)	79.0 (74.5–83.4)	70.6 (65.5–75.8)	27.9 (21.8–33.9)	52.3 (45.6–58.9)
	≥ 75	23.9 (19.4–28.5)	32.2 (26.1–38.4)	70.2 (60.7–79.7)	64.7 (55.0–74.5)	27.5 (14.4–40.5)	57.5 (44.8–70.2)
Residency	Rural	11.0 (10.0–12.0)	22.9 (21.5–24.2)	70.6 (66.1–75.1)	62.8 (58.1–67.5)	25.5 (20.0–31.0)	46.3 (40.1–52.4)
	Urban	15.2 (14.3–16.1)	25.4 (24.3–26.6)	73.9 (71.1–76.8)	65.6 (62.5–68.7)	28.5 (25.0–32.0)	52.5 (48.5–56.5)
Wealth index (quintiles)	First (Poorest)	13.3 (11.9–14.7)	26.4 (24.1–28.7)	72.5 (67.7–77.4)	63.8 (58.6–69.0)	28.9 (22.6–35.2)	49.4 (42.2–56.6)
	Second	15.5 (13.7–17.2)	24.1 (22.0–26.1)	72.0 (66.4–77.6)	63.9 (58.0–69.8)	28.5 (21.7–35.4)	52.5 (44.7–60.3)
	Third	13.5 (12.1–14.9)	24.9 (23.0–26.9)	75.5 (70.5–80.4)	66.4 (60.8–71.9)	21.2 (15.9–26.5)	42.1 (35.3–48.8)
	Fourth	14.6 (12.9–16.2)	26.0 (24.0–28.1)	69.9 (63.6–76.1)	62.1 (55.8–68.4)	27.7 (20.7–34.6)	49.6 (42.3–57.0)
	Fifth (Wealthiest)	14.1 (12.1–16.1)	22.8 (20.4–25.1)	77.0 (71.4–82.6)	70.3 (63.8–76.9)	31.7 (23.5–39.8)	58.8 (49.4–68.3)
Education (years of schooling)	0	21.4 (19.6–23.2)	29.1 (26.9–31.3)	74.6 (70.5–78.8)	66.9 (62.3–71.5)	24.8 (20.1–29.5)	47.6 (42.1–53.2)
	1–6	18.6 (17.1–20.1)	25.9 (24.1–27.7)	78.4 (74.6–82.1)	69.9 (65.9–73.9)	27.5 (22.7–32.2)	49.0 (43.6–54.4)
	7–11	11.3 (9.7–12.8)	25.3 (23.2–27.4)	70.8 (64.6–77.0)	63.1 (56.4–69.8)	28.0 (19.0–37.0)	51.7 (41.8–61.5)
	≥ 12	10.0 (8.8–11.2)	22.3 (20.7–23.9)	67.4 (61.6–73.1)	58.6 (52.5–64.7)	30.9 (24.0–37.8)	56.7 (48.4–64.9)
Basic insurance	No	9.4 (7.2–11.7)	23.4 (20.2–26.7)	57.4 (44.1–70.6)	49.7 (36.9–62.4)	20.3 (9.7–30.9)	45.7 (29.1–62.4)
	Yes	14.6 (13.9–15.4)	25.0 (24.0–25.9)	74.3 (71.8–76.7)	66.0 (63.3–68.6)	28.2 (25.1–31.3)	51.4 (47.8–54.9)
Complementary insurance	No	11.5 (10.7–12.3)	24.0 (22.8–25.1)	67.4 (64.0–70.8)	58.0 (54.5–61.5)	30.6 (26.3–34.8)	51.4 (46.7–56.1)
	Yes	20.6 (19–22.2)	26.9 (25.1–28.7)	81.0 (77.6–84.4)	74.3 (70.4–78.1)	25.1 (20.9–29.4)	50.8 (45.6–56.0)
Total		14.2 (13.4–14.9)	24.8 (23.9–25.7)	73.3 (70.8–75.7)	65.0 (62.4–67.7)	28.0 (25.0–31.0)	51.4 (47.9–54.8)

The national prevalence of prediabetes was 24.8% (23.9–25.7), with a significantly higher prevalence in males (26.4% [24.9–27.8]) compared to females (23.5% [22.3–24.8]). Prediabetes was significantly more prevalent among the population in urban areas (25.4% [24.3–26.6]) rather than in rural population (22.9% [21.5–24.2]). Among WI quintiles, the poorest population had the highest prediabetes rates (26.4% [24.1–28.7]) and wealthiest population had the least rates (22.8% [20.4–25.1]) (Table 1). Among provinces, prevalence of prediabetes was lowest in Zanjan (16.1% [13.5–18.6]) and highest in Gilan (31.1% [25.0–37.3]) (Fig. 1B).

### Diabetes awareness

Overall, 73.3% (70.8–75.7) of the population with diabetes were aware of their condition for both sexes. The awareness estimation was 69.0% (65.0–73.0) among males and significantly higher as 76.4% (73.3–79.5) among females. Awareness was higher in urban residents (73.9% [71.1–76.8]) compared to those in rural areas (70.6% [66.1–75.1]). Regarding the WI, the wealthiest population with diabetes had the highest disease awareness (77.0% [71.4–82.6]). Also, DM awareness was significantly higher in patients having basic and complementary insurance (Table 1, Fig. 1C).

### Diabetes treatment coverage and glycemic control

The overall DM treatment coverage was estimated at 65.0% (62.4–67.7) for both sexes, 60.4% (56.1–64.8) in males and significantly higher as 68.4% (65.1–71.7) in females. Treatment coverage was higher in older population, in urban areas, wealthier population, and those with lesser education. Also, having any kind of basic or complementary insurance raised DM treatment coverage significantly (Table 1, Fig. 1D). Good glycemic control was achieved in 28.0% (25.0–31.0) of all patients with diabetes, with higher statistics in females (29.0% [25.2–32.8]) compared to males (26.4% [21.4–31.4]). Fair glycemic control was achieved in about 51.4% (47.9–54.8) of all with diabetes, also higher in females (52.5% [48.4–56.7]) compared to males (49.5% [43.4–55.6]). Both good and fair glycemic control were happened more in younger, urban resided, wealthier, and higher educated population with diabetes (Table 1, Fig. 1E,F).

### WHO coverage targets

Compared to the 80% target of diabetes diagnosis, this study estimated 73.3% success in diagnosing the condition. Good glycemic control among those previously diagnosed with DM, was achieved only in 36.7% and fair glycemic control was achieved in 58.4%. Also, blood pressure in previously diagnosed patients was controlled to less than 140/90 in 55.2% and to less than 130/80 in only 28.5%. Statins were used by 50.1% of patients with diabetes in this survey (Table 2).

**Table 2:** Compliance with World Health Organization global coverage targets for diabetes in population with diabetes in Iran STEPS Survey 2021.

Target	WHO goal	Iran STEPS survey 2021 (prevalence% (95% confidence interval))
1. Diabetes diagnosis	80%	73.3 (70.8–75.7)
2. Good glycemic control	80%	HbA1c < 7%: 36.7 (33.8–39.5)
		HbA1c < 8%: 58.4 (55.5–61.4)
3. Good blood pressure control	80%	BP < 140/90: 55.2 (52.2–58.1)
		BP < 130/80: 28.5 (25.9–31)
4. Statin use	60%	50.1 (47.2–52.9)
5. Access to insulin and blood glucose self-monitoring in type 1 diabetes	100%	NA

### Distribution of antihyperglycemic treatment

Among various antihyperglycemic agents used by patients among all with DM, the prevalence of non-insulin medication was 60.5% (57.8–63.2) and the most prescribed group of medications was Biguanides (49.6% [46.8–52.4]). Among the population with DM, the prevalence of insulin use was 12.1% (10.4–13.8) with long-acting insulin with the highest use (3.4% [2.6–4.2]). Also, patients reported the utilization of pen Insulin at about 10.1% (8.6–11.6). Including both insulin and non-insulin drugs, the prevalence of combination treatment with two drugs was 36.9% (34.2–39.6), with three drugs was 16.5% (14.6–18.4), and with four drugs was 7.4% (5.9–8.8). Also, the prevalence of herbal medicine use was about 13.0% (10.9–15.1) (Table 3).

**Table 3:** Prevalence of categories of antihyperglycemic treatment in all with diabetes patients in Iran STEPS Survey 2021.

Antihyperglycemic treatment	Sub groups	Prevalence% (95% confidence interval)		
Categories		Male	Female	Both
Non-insulin drugs	All	56.4 (52.0–60.9)	63.4 (60.1–66.8)	60.5 (57.8–63.2)
	Biguanides	44.9 (40.4–49.4)	53 (49.5–56.5)	49.6 (46.8–52.4)
	Sulfonylureas	19.7 (16.5–23.0)	21.3 (18.5–24.1)	20.6 (18.5–22.8)
	Meglitinides	0.7 (0.2–1.2)	0.7 (0.3–1.2)	0.7 (0.4–1.1)
	Thiazolidinediones	0.9 (0.4–1.5)	0.8 (0.4–1.3)	0.9 (0.5–1.2)
	Alpha-glucosidase inhibitors	1.4 (0.4–2.4)	1.8 (0.9–2.8)	1.6 (1.0–2.3)
	Dipeptidyl peptidase-4 inhibitor	2.1 (1.1–3.2)	2.6 (1.4–3.8)	2.4 (1.6–3.2)
	Glucagon-like peptide-1 receptor agonists	0.1 (0.0–0.4)	0.3 (0.0–0.7)	0.3 (0.0–0.5)
	Other	2.0 (0.4–3.5)	3.3 (1.4–5.2)	2.8 (1.5–4.0)
Insulin	All	9.6 (7.2–12.0)	14.0 (11.6–16.3)	12.1 (10.4–13.8)
	Rapid-acting insulin	2.4 (1.0–3.8)	3.2 (2.0–4.5)	2.9 (2.0–3.8)
	Short-acting insulin	2.1 (1.1–3.1)	3.4 (2.1–4.7)	2.9 (2.0–3.7)
	Intermediate-acting insulin	1.5 (0.7–2.3)	2.7 (1.4–3.9)	2.2 (1.4–3.0)
	Long-acting insulin	3.4 (2.1–4.8)	3.3 (2.4–4.3)	3.4 (2.6–4.2)
	Mixed insulin	2.6 (1.2–3.9)	4.4 (3.0–5.9)	3.6 (2.6–4.6)
Pen insulin		8.5 (6.3–10.8)	11.2 (9.2–13.2)	10.1 (8.6–11.6)
Combination treatments (any insulin and non-insulin)	1 drug (no combination)	41.2 (36.8–45.6)	34.2 (30.8–37.6)	37.1 (34.4–39.9)
	2 drugs	37.4 (32.8–42.0)	36.5 (33.3–39.7)	36.9 (34.2–39.6)
	3 drugs	13.7 (11.2–16.2)	18.5 (15.9–21.2)	16.5 (14.6–18.4)
	4 drugs	5.9 (3.9–7.9)	8.4 (6.4–10.4)	7.4 (5.9–8.8)
	5 drugs	1.3 (0.6–2.1)	2.1 (1.3–2.9)	1.8 (1.2–2.3)
	6 drugs	0.4 (0.0–0.9)	0.3 (0.0–0.7)	0.4 (0.1–0.7)
Herbal medicine	All	12.0 (8.6–15.4)	13.7 (11.0–16.3)	13.0 (10.9–15.1)
	Alone	2.5 (0.4–4.6)	2.5 (0.7–4.3)	2.5 (1.1–3.8)
	In combination with any other types of drugs (Insulin and non-insulin)	9.5 (6.7–12.4)	11.2 (9.0–13.3)	10.5 (8.8–12.2)

### Other findings

The estimated number of population with DM in Iran population aged ≥ 25 years old was about 6,935,886 (6,580,648–7,291,123) for both sexes nationally, and higher among females and urban residents. The estimated number of population with prediabetes in Iranian adults aged ≥ 25 years was estimated about 12,148,229 (11,692,071–12,604,387), and higher among males and urban residents. Quality of life in patients with DM showed more severe concern with pain/discomfort (debilitated: 10.1% [8.6–11.6]), and anxiety/depression (debilitated: 8.8% [7.4–10.1]) areas.

Among all participants with DM in this study, the prevalence of having a glucometer was 47.8% (45.0–50.5), and this percentage was higher among older patients, those living in cities, wealthier population, and those with any kind of insurance. The estimated mean onset age of DM based on self-reported values by participants was 47.3 (46.4–48.1) nationally for both sexes, with onset of disease mainly in the fifth decade of life in most of the population. The estimated prevalence of hypoglycemic events in the past two weeks among patients with DM in this study was 19.6% (16.9–22.4) for both sexes, and the events were more prevalent in the younger, rural resident, and those with lower WI and education. Among all participants in the Iran STEPS Survey 2021, the prevalence of a positive family history of DM was 31.7% (31.1–32.4), while among patients with DM this measure was significantly higher as 56.4% (53.6–59.3). More details on results of study are provided in the Supplementary Appendix.

## Discussion

The study investigated the most recent epidemiology of DM and prediabetes in Iran and highlighted remarkable findings. About one out of seven of the adult population above 25 years of age suffer from diabetes. Also, about one-fourth of adult population without diabetes, had prediabetes. About three quarters of the participants with DM were aware of their condition, and a lesser proportion were under treatment; however, good glycemic control was achieved in about one fourth of the patients with diabetes. The global diabetes coverage targets on DM diagnosis and statin use were near to optimal; however, glycemic and blood pressure control were much way sub-optimal. HRQoL was majorly affected by DM in these patients by a remarkable report of pain/discomfort and anxiety/depression.

The primary objective of this study was to report the most recent epidemiology of DM in Iran and its provinces. The latest Global Burden of Diseases (GBD) 2019 estimations for Iran reported an age-standardized rate of DM of about 6702 (95% uncertainty interval 6079–7361) prevalent cases per 100,000 population with nearly doubling during the past 30 years^20^. Previous Iran STEPS Survey 2016 reported the prevalence of DM at 10.6% (10.0–11.1) according to either high FPG or self-report of taking at least one anti-diabetes medication; however, based on other definitions, the prevalence of DM reached 14.2% (13.6–14.8)^10^. The prevalence of prediabetes in previous STEPS was 16.6% (15.9–17.2) based on 100 ≤ FPG < 126 mg/dL^10^. In a nationally representative cross-sectional survey of Iranian adults aged 35–70 years as part of the PERSIAN Cohort Study, the sex- and age-standardized prevalence of DM was 15.0% (12.6–17.3), and prediabetes was 25.4% (18.6–32.1)^9^. Older national estimations reported the prevalence of DM and impaired fasting glucose about 8.7% (7.4–10.2) and 9.2% (7.9–10.7) from a national survey on the 25–64 years popualtion in 2007^21^, and 11.4% (9.9–12.9) and 14.6% (12.4–16.8) from a national survey on the 25–70 years population in 2011^22^, respectively.

The other finding of this study was the prevalence of prediabetes in abort a quarter of population. As a metabolic state with a high chance of conversion to DM in the future, prediabetes is of high importance^23^. As one of the major metabolic risk factors and precursors of NCDs, high FPG ranked second investigating the NCDs’ deaths attributable to risks, and ranked third investigating the NCDs’ DALYs attributable to risks, and the burden grew more than behavioural factors like smoking and dietary risks in the past three decades^20^. Variation of prediabetes based on different guidelines is a major issue and leads to remarkable differences in estimations; therefore, the diagnostic criteria for prediabetes evaluation need more investigations^24^.

This study found a higher prevalence of DM among females and a higher prevalence of prediabetes among males. The paradoxical sex difference in the prevalence of DM and prediabetes were consistent with previous STEPS 2016 Survey and other national estimations^9,10,21,22^. It is evident in literature although impaired fasting glucose is more prevalent in males, impaired glucose tolerance is more prevalent in females. Several hypotheses have been proposed on the role of sex hormones, specifically estrogen and its changes after menopause, differences in insulin sensitivity in the two sexes, and the most important, the different pathophysiology of DM in males and females^25^. Due to the distinct inherent metabolic characteristics of impaired fasting glucose and impaired glucose tolerance which is more susceptible to DM^26^, it is suggested that the first state be more complementary in evaluating the chance of progression toward DM and not as a definite diagnostic tool^27^.

The current study showed that most patients with DM were aware of their condition. Previous national study found awareness about 79.6% among patients with DM and with a higher statistics in females^9^. Considering the impact of other factors on DM awareness, a study from Iran showed that lower education was significantly associated with lower awareness leading to lower self-care and higher adverse outcomes of DM^28^. Results of a prospective cohort from the west of Iran that estimated DM awareness of about 78.5% among those with diabetes, suggested that a significant proportion of the high awareness could be attributed to the integration of DM care into the primary healthcare (PHC) system^29^.

Although more than half of the population with DM was under treatment in the current investigation, only about half of the target population had fair glycemic control and only a quarter had good glycemic control. The STEPS Survey 2016 reported that 52.1% (49.4–54.7) of patients with self-reported DM were under strict glycemic control^10^. Another national study reported glycemic control in about 41.2% of patients with DM receiving treatment^9^. An investigation of a national survey conducted in 2005 in Iran showed 39.2% (37.7–40.7) of individuals with DM received treatment, and this coverage could lower mean FPG significantly higher in rural areas of Iran where the main health provider is PHC workers known as Behvarz workers, suggesting the effectiveness of Iran PHC in prevention and management of NCDs and related risk factors^30^. A review of the literature revealed that the quality of diabetes care had improved gradually in the past decades in Iran as the proportion of undiagnosed DM cases decreased and diabetes medications became more affordable^13^. In the current study, about one-sixth of the patients with DM were using insulin, and a large proportion of this sample reported using pen insulin. Although the pen insulin is easier to use, a study revealed more expensive pen insulin was not associated with better glycemic control and other related adverse outcomes of DM in Iran^31^.

One of the main findings of this study was disparities favoring a higher DM prevalence and worse disease awareness and care among the less fortunate population. The effect of socioeconomic factors on the prevalence and care of DM and prediabetes is a significant issue in Iran, as a systematic review of socioeconomic inequalities and DM reported a higher prevalence of disease and its complications in a population with poorer socioeconomic status^32^. The impact of education level, occupation, and income on DM prevalence and outcomes has been consistent among populations residing in high-, middle-, and low-income countries showing the strength of this association regardless of other confounders^33^.

This study found a high impact of pain/discomfort and depression/anxiety related to DM on patients quality of life. This finding was consistent with a similar nationwide survey on DM patients in Iran reporting an overall relatively poor quality of life^34^. A meta-analysis of studies on HRQoL in Iran revealed that patients with DM have a moderate quality of life, and improvements, especially in physical aspects, were recommended^35^. Consistent with the findings of this survey, literature shows that depression and anxiety among patients with DM in Iran are alarming and relatively higher in comparison to other countries, and this issue needs specific attention and actions to tackle^36^.

As a LMIC with a huge burden of DM, the results of this study in Iran are comparable with similar countries. Governments benefit from the STEPS framework to study diabetes epidemiology; however, the results vary due to different characteristics of populations and measurement tools. Therefore, inspecting pooled analyses from countries might be more informative. A pooled analysis of 55 nationally representative surveys in LMICs, noted that fewer than one in ten patients with DM in these countries receive guideline-based treatment coverage for diabetes^6^. Although DM screening, diagnosis, and treatment are vital to control this disease, a pooled analysis of data from 67 LMICs showed that leveraging blood pressure control and statin coverage contribute more significantly to the management of DM and its complications^37^. Incorporating such pooled results for national inferences would benefit health policymakers in LMICs.

The current study was successful in investigating the DM care cascade and compliance of diabetes coverage targets. Our findings highlighted although majority of patients with DM are diagnosed in Iran, the glycemic control was submopitmal. Also, hypertension as a major comorbidity in patients with DM, was poorly controlled and needs attention to reduce complications. Comparing the findings on WHO targets with recently published literature shows gaps in diabetes care in Iran, as a secondary analysis for WHO targets, reported 80–90% DM diagnosis rate, more than 50% patients with good glycemic control and more than 80% with fair glycemic control, 50% achievement in strict blood pressure control and 70% control in the higher cut-off, and over 60% statin use among patients with diabetes in the United States for a pooled data of 1999–2018^38^. Further investigations based on these targets are recommended to study different areas with tangible goals using these simple targets.

In recent decades, Iran has tackled the DM epidemic through various screening and treatment plans and mainly by expanding the PHC services^8,20^. Also, by establishing the national action plan for NCDs prevention and control, the endeavours were focused on DM as one of the top burdensome NCDs in Iran^39^. Further qualitative research has shown the challenges of DM prevention and control in Iran as six themes of referral system shortages, human resources, infrastructure, cultural problems, access, and intersectoral coordination issues^40^. Insulin, one of the fundamentals of DM control, has faced shortages in supply in the past couple of years, and the health system is struggling to provide this vital medication to take the DM epidemic under control^41^. Improvements in health system management and expanded national action plans are needed to address the growing prevalence and burden of DM in Iran.

Recruiting a robust methodology and a nationally representative sample in Iran STEPS Survey 2021 while the COVID-19 pandemic was raging in country, was the most remarkable strength of this survey^16^. However, the study had some limitations. Interruptions in data and sample collection due to the COVID-19 pandemic brought up some challenges in generating survey results^16^. As a limitation of this study, the blood sample for measuring the FPG and HbA1C levels was taken only once, and more samples were not available for a two-step assessment. Also, the self-reports in the first step of survey could be biased and was another limitation. The biased self-report is a major limitation in assessing DM treatment that could bias the presented results on the treatment coverage and type of anti-hyperglycemic agents. Unfortunately, at this time there is no validated tool and database to gauge the validity and accuracy of self-reports on DM treatment in Iranian healthcare context; however, recent advances in using the claims and health insurance data to validate such data have been made and there is a hope to improve the results of surveys like this study in future. As another main outcome of this study, assessing the prevalence of hypoglycemia based solely on self-reports can be challenging and may introduce potential biases including underreporting, recall, and misinterpretation of signs biases which should be considered in interpreting the results on this major complication of DM and its treatment. However, the investigators tried to reduce the biases and obstacles by adherence to the validated WHO STEPS framework^17^.

## Conclusion

Despite the growing prevalence of DM and prediabetes in Iran, awareness about this disease, treatment coverage, and glycemic control patterns overall known as care cascade have not improved adequately, indicating an alarming state of diabetes care for now and future. The presence of various demographic, socioeconomic, and geographic disparities in the prevalence of DM and prediabetes were the other signs of the health system’s weakness in providing equitable and accessible healthcare for all in Iran. Major revisions on the national diabetes prevention and control program are essential to curb the burden of DM and its complications on individuals and the health system.

### Supplementary Information

Supplementary Information.

## Data Availability

The datasets generated and/or analysed during the current study are not publicly available due to the restrictions set by the funder of study, I.R. Iran’s National Institute of Health Research, but are available from the corresponding author on reasonable request.
