# Supplementary material for: Evaluation of the diabetes care cascade and compliance with WHO global coverage targets in Iran based on STEPS survey 2021
Source: Sci Rep. 2023 Aug 19;13:13528. doi: 10.1038/s41598-023-39433-7 (PMC10439917; doi:10.1038/s41598-023-39433-7)

Supplementary appendix to "Evaluation of the Diabetes Care Cascade and Compliance with WHO Global Coverage Targets in Iran based on STEPS Survey 2021".

This appendix contains supplementary results.

Table of contents

Supplementary table 1. Prevalence of diabetes by population characteristics in Iran STEPS Survey 2021....3

Supplementary table 2. Prevalence of diabetes by sex and residency in provinces of Iran in STEPS Survey 2021. 4

Supplementary table 3. Prevalence of prediabetes by population characteristics in Iran STEPS Survey 2021. 5

Supplementary table 4. Prevalence of prediabetes by sex and residency in provinces of Iran in STEPS Survey 2021. 6

Supplementary table 5. Prevalence of diabetes awareness among all with diabetes in Iran STEPS Survey 2021. 7

Supplementary table 6. Prevalence of diabetes treatment coverage among all with diabetes in Iran STEPS Survey 2021....8

Supplementary table 7. Prevalence of good (HbA1C<7%) and fair (HbA1C<8%) glycemic control among all with diabetes in Iran STEPS Survey 2021. 9

Supplementary table 8. Estimated number of the population with diabetes and prediabetes based on Iran STEPS Survey 2021. 10

Supplementary table 9. Health-related quality of life assessment in all diabetic patients included in Iran STEPS Survey 2021. 11

Supplementary table 10. Prevalence of owning a glucometer by patients with diabetes included in Iran STEPS Survey 2021. 12

Supplementary table 11. Mean onset age of diabetes mellitus among patients included in Iran STEPS Survey 2021....13

Supplementary table 12. Prevalence of hypoglycemic event in past two weeks in patients with diabetes included in Iran STEPS Survey 2021. 14

Supplementary table 13. Prevalence of positive family history of diabetes among all participants in Iran STEPS Survey 2021....15

Supplementary table 14. Prevalence of positive family history of diabetes mellitus among all participants with diabetes included in Iran STEPS Survey 2021. 16

Supplementary figure 1. Subnational prevalence of (A) diabetes, and (B) prediabetes among provinces in Iran STEPS Survey 2021. 17

Supplementary figure 2. Prevalence of (A) diabetes, and (B) prediabetes in different age categories of each sex in Iran STEPS Survey 2021. 18

Supplementary table 1. Prevalence of diabetes by population characteristics in Iran STEPS Survey 2021.

**Diabetes**		**Prevalence% (95% confidence interval)**		
**Categories**	**Subcategories**	**Male**	**Female**	**Both**
Age	25 - 34	2.3 (1.2–3.4)	2.0 (1.2–2.7)	2.1 (1.5–2.7)
	35 - 44	5.7 (4.2–7.2)	5.7 (4.2–7.2)	5.7 (4.7–6.8)
	45 - 54	14.9 (12.3–17.4)	16.7 (14.6–18.8)	15.9 (14.3–17.5)
	55 - 64	24.3 (20.9–27.7)	28.9 (26.1–31.8)	26.8 (24.7–29.0)
	65 - 74	23.2 (19.4–27.0)	30.5 (26.7–34.2)	27.0 (24.4–29.7)
	≥75	25.0 (18.7–31.2)	22.6 (16.1–29.0)	23.9 (19.4–28.5)
Residency	Rural	9.0 (7.6–10.4)	12.6 (11.2–14.0)	11.0 (10.0–12.0)
	Urban	14.9 (13.5–16.3)	15.4 (14.2–16.6)	15.2 (14.3–16.1)
Wealth Index (quintiles)	First (Poorest)	9.9 (7.9–12.0)	15.8 (13.8–17.7)	13.3 (11.9–14.7)
	Second	14.4 (11.9–17.0)	16.2 (13.9–18.6)	15.5 (13.7–17.2)
	Third	12.6 (10.6–14.7)	14.3 (12.3–16.3)	13.5 (12.1–14.9)
	Fourth	13.9 (11.6–16.3)	15.1 (12.8–17.5)	14.6 (12.9–16.2)
	Fifth (Wealthiest)	16.1 (12.9–19.2)	12.2 (9.9–14.4)	14.1 (12.1–16.1)
Education (years of schooling)	0	16.6 (13.6–19.6)	23.3 (21.1–25.5)	21.4 (19.6–23.2)
	1-6	17.2 (15–19.5)	19.5 (17.6–21.5)	18.6 (17.1–20.1)
	7-11	10.7 (8.6–12.9)	11.8 (9.7–13.9)	11.3 (9.7–12.8)
	≥12	12.2 (10.4–14.0)	7.9 (6.4–9.4)	10.0 (8.8–11.2)
Basic insurance	No	8.7 (5.6–11.7)	10.2 (6.8–13.6)	9.4 (7.2–11.7)
	Yes	14.0 (12.8–15.2)	15.1 (14.1–16.1)	14.6 (13.9–15.4)
Complementary insurance	No	10.9 (9.7–12.1)	12.0 (11.0–13.0)	11.5 (10.7–12.3)
	Yes	19.7 (17.2–22.3)	21.3 (19.2–23.4)	20.6 (19–22.2)
**Total**		13.5 (12.3–14.6)	14.7 (13.8–15.7)	14.2 (13.4–14.9)

Supplementary table 2. Prevalence of diabetes by sex and residency in provinces of Iran in STEPS Survey 2021.

**Diabetes**	**Prevalence% (95% confidence interval)**				
**Location**	**Male**	**Female**	**Rural**	**Urban**	**Total**
Alborz	5.5 (2.6–8.4)	11.9 (7.4–16.4)	11.8 (11.8–11.8)	8.6 (8.6–8.6)	9.1 (6.2–12.0)
Ardebil	8.8 (5.4–12.2)	14.3 (9.2–19.5)	10.5 (5.6–15.5)	12.2 (8.0–16.3)	11.7 (8.4–15.0)
Bushehr	11.4 (7.9–14.8)	12.6 (9.4–15.8)	8.8 (5.3–12.3)	13.2 (10.3–16.1)	11.9 (9.6–14.2)
Chahar mahall and Bakhtiari	3.8 (2.1–5.6)	8.5 (6.2–10.9)	5.9 (3.4–8.4)	6.2 (4.2–8.2)	6.2 (4.6–7.7)
East Azarbaijan	9.1 (3.4–14.8)	6.0 (3.6–8.4)	5.3 (2.6–7.9)	8.1 (4.2–12.1)	7.3 (4.4–10.2)
Esfahan	9.4 (7.0–11.8)	9.4 (7.4–11.3)	7.0 (3.9–10.1)	9.4 (7.8–11.1)	9.2 (7.7–10.7)
Fars	8.6 (5.9–11.3)	12.7 (10.0–15.4)	8.7 (5.5–11.8)	11.2 (8.9–13.5)	10.6 (8.8–12.5)
Gilan	13.3 (5.8–20.8)	14.3 (8.5–20.1)	11.7 (5.2–18.3)	14.9 (8.6–21.2)	13.8 (9.2–18.4)
Golestan	9.6 (6.4–12.8)	12.4 (9.3–15.5)	8.1 (5.1–11.1)	13.3 (10.2–16.4)	11.0 (8.8–13.2)
Hamadan	7.0 (4.2–9.8)	6.4 (4.4–8.4)	3.7 (1.8–5.5)	7.8 (5.6–10.1)	6.6 (4.9–8.4)
Hormozgan	11.5 (6.4–16.7)	13.1 (8.2–18.1)	7.0 (3.6–10.5)	20.0 (13.6–26.5)	12.4 (8.7–16.0)
Ilam	7.7 (3.8–11.6)	10.7 (6.9–14.5)	7.2 (3.2–11.3)	10.3 (6.6–13.9)	9.3 (6.5–12.0)
Kerman	7.7 (4.0–11.4)	10.5 (6.0–15.0)	5.3 (2.8–7.9)	11.9 (7.3–16.5)	9.3 (6.4–12.3)
Kermanshah	6.1 (3.1–9.1)	5.8 (2.7–8.9)	5.8 (2.0–9.5)	5.7 (3.2–8.3)	5.8 (3.6–8.1)
Khuzestan	13.9 (9.6–18.3)	18.4 (14.8–22.0)	16.1 (10.5–21.6)	16.4 (13.0–19.8)	16.3 (13.4–19.2)
Kohgiluyeh and Buyer Ahmad	7.2 (4.2–10.3)	9.1 (5.7–12.6)	7.9 (4.3–11.6)	9.2 (5.8–12.6)	8.6 (6.2–11.0)
Kordestan	10.2 (7.0–13.5)	12.3 (8.9–15.6)	5.9 (2.7–9.1)	13.5 (10.4–16.7)	11.6 (9.2–14.0)
Lorestan	9.1 (5.3–13.0)	11.4 (8.7–14.2)	8.1 (4.9–11.3)	11.7 (8.6–14.7)	10.5 (8.2–12.7)
Markazi	9.6 (4.7–14.5)	8.8 (6.3–11.3)	4.4 (1.6–7.1)	10.2 (7.0–13.4)	8.9 (6.4–11.4)
Mazandaran	8.9 (6.0–11.9)	16.1 (12.4–19.8)	12.5 (8.9–16.0)	12.9 (9.6–16.3)	12.9 (10.4–15.4)
North Khorasan	9.3 (5.8–12.7)	7.7 (5.0–10.3)	6.1 (3.2–9.0)	9.8 (6.7–12.8)	8.2 (6.1–10.3)
Qazvin	9.3 (5.6–12.9)	12.9 (8.0–17.9)	5.0 (1.6–8.5)	13.4 (9.4–17.5)	11.3 (8.0–14.5)
Qom	10.9 (6.8–15.0)	10.2 (6.7–13.7)	18.6 (11.8–25.4)	10.2 (7.5–12.9)	10.6 (7.9–13.2)
Razavi Khorasan	10.0 (7.3–12.6)	10.5 (8.3–12.7)	8.4 (5.7–11.2)	10.9 (8.7–13.0)	10.2 (8.5–11.9)
Semnan	10.3 (6.5–14.1)	9.8 (6.9–12.8)	13.2 (8.7–17.7)	8.8 (6.2–11.3)	9.7 (7.5–12.0)
Sistan and Baluchestan	10.9 (6.5–15.3)	14.2 (10.4–17.9)	11.6 (8.1–15.2)	12.9 (8.6–17.1)	12.9 (10.0–15.7)
South Khorasan	7.7 (4.2–11.1)	9.4 (6.3–12.5)	4.9 (2.6–7.2)	12.0 (8.1–15.8)	9.3 (6.7–11.9)
Tehran	13.5 (10.4–16.6)	11.0 (8.5–13.5)	10.7 (6.1–15.2)	12.2 (10.0–14.4)	12.3 (10.2–14.4)
West Azarbaijan	9.7 (6.3–13.1)	8.8 (5.9–11.6)	5.5 (2.5–8.6)	10.1 (7.3–12.9)	9.1 (6.8–11.4)
Yazd	9.9 (6.0–13.8)	13.7 (7.6–19.8)	6.9 (1.8–12.1)	13.2 (8.3–18.1)	12.2 (8.1–16.2)
Zanjan	7.3 (4.8–9.8)	10.5 (7.9–13.0)	7.3 (4.0–10.5)	9.8 (7.6–12.1)	9.0 (7.2–10.8)
**National**	13.5 (12.3–14.6)	14.7 (13.8–15.7)	11.0 (10.0–12.0)	15.2 (14.3–16.1)	14.2 (13.4–14.9)

Supplementary table 3. Prevalence of prediabetes by population characteristics in Iran STEPS Survey 2021.

**Prediabetes**		**Prevalence% (95% confidence interval)**		
**Categories**	**Sub categories**	**Male**	**Female**	**Both**
Age	25 - 34	18.6 (15.6–21.6)	13.4 (11.5–15.3)	15.7 (14.0–17.4)
	35 - 44	26.9 (23.8–30.1)	20.5 (18.4–22.6)	23.2 (21.4–25.0)
	45 - 54	28.0 (25.0–30.9)	27.4 (24.9–29.9)	27.6 (25.7–29.5)
	55 - 64	30.1 (26.9–33.4)	27.6 (24.6–30.7)	28.8 (26.5–31.0)
	65 - 74	28.7 (24.1–33.4)	31.2 (27.0–35.3)	30.0 (26.9–33.1)
	≥75	28.6 (22.7–34.5)	36.9 (25.6–48.1)	32.2 (26.1–38.4)
Residency	Rural	23.7 (21.8–25.7)	22.1 (20.3–23.9)	22.9 (21.5–24.2)
	Urban	27.2 (25.4–29.0)	24.0 (22.5–25.5)	25.4 (24.3–26.6)
Wealth Index (quintiles)	First (Poorest)	25.4 (22.3–28.4)	27.1 (23.8–30.3)	26.4 (24.1–28.7)
	Second	25.0 (21.9–28.1)	23.4 (20.7–26.1)	24.1 (22.0–26.1)
	Third	27.8 (24.8–30.8)	22.3 (19.9–24.7)	24.9 (23.0–26.9)
	Fourth	27.4 (24.4–30.3)	24.8 (22.0–27.5)	26.0 (24.0–28.1)
	Fifth (Wealthiest)	25.3 (21.6–28.9)	20.3 (17.3–23.3)	22.8 (20.4–25.1)
Education (years of schooling)	0	28.8 (24.9–32.6)	29.3 (26.6–31.9)	29.1 (26.9–31.3)
	1-6	26.3 (23.8–28.8)	25.6 (23.2–28.1)	25.9 (24.1–27.7)
	7-11	25.8 (22.9–28.7)	24.8 (21.8–27.8)	25.3 (23.2–27.4)
	≥12	26.2 (23.8–28.6)	18.5 (16.5–20.4)	22.3 (20.7–23.9)
Basic insurance	No	24.2 (19.8–28.7)	22.6 (17.9–27.3)	23.4 (20.2–26.7)
	Yes	26.6 (25.1–28.2)	23.6 (22.4–24.9)	25.0 (24.0–25.9)
Complementary insurance	No	25.1 (23.4–26.8)	23.0 (21.6–24.5)	24.0 (22.8–25.1)
	Yes	29.5 (26.7–32.3)	24.9 (22.6–27.2)	26.9 (25.1–28.7)
**Total**		26.4 (24.9–27.8)	23.5 (22.3–24.8)	24.8 (23.9–25.7)

Supplementary table 4. Prevalence of prediabetes by sex and residency in provinces of Iran in STEPS Survey 2021.

**Prediabetes**	**Prevalence% (95% confidence interval)**				
**Location**	**Male**	**Female**	**Rural**	**Urban**	**Total**
Alborz	29.5 (18.5–40.5)	18.5 (12.3–24.8)	8.4 (8.4–8.4)	23.8 (23.8–23.8)	23.1 (17.2–28.9)
Ardebil	17.2 (12.0–22.4)	21.4 (15.8–27.1)	16.0 (9.9–22.1)	20.8 (15.9–25.8)	19.6 (15.6–23.5)
Bushehr	22.0 (16.6–27.4)	15.1 (11.4–18.8)	16.0 (11.0–21.0)	19.0 (15.0–23.1)	18.2 (15.0–21.3)
Chahar mahall and Bakhtiari	18.9 (13.4–24.5)	14.3 (10.5–18.1)	11.6 (7.0–16.3)	18.7 (14.4–23.0)	16.3 (13.0–19.5)
East Azarbaijan	13.8 (8.9–18.6)	18.8 (13.7–23.9)	12.9 (8.4–17.4)	17.6 (12.9–22.3)	16.3 (12.8–19.9)
Esfahan	15.9 (11.1–20.7)	16.7 (13.5–20.0)	11.3 (6.3–16.3)	16.8 (13.8–19.9)	16.3 (13.5–19.0)
Fars	24.1 (19.5–28.6)	22.8 (18.7–26.8)	25.5 (20.3–30.8)	23.5 (19.8–27.3)	23.6 (20.6–26.7)
Gilan	37.6 (26.3–48.9)	27.4 (20.2–34.6)	31.0 (22.2–39.8)	31.5 (22.9–40.0)	31.1 (25.0–37.3)
Golestan	32.8 (27.2–38.4)	24.2 (19.7–28.7)	24.6 (19.5–29.8)	31.6 (26.4–36.8)	28.4 (24.8–32.0)
Hamadan	16.7 (11.6–21.7)	16.6 (12.3–20.8)	18.0 (12.3–23.7)	15.9 (11.8–19.9)	16.4 (13.2–19.6)
Hormozgan	33.2 (25.0–41.3)	25.4 (19.2–31.6)	29.4 (23.0–35.9)	27.4 (18.6–36.3)	28.3 (23.4–33.2)
Ilam	20.1 (14.1–26.2)	21.1 (14.5–27.7)	20.8 (13.1–28.5)	19.9 (14.4–25.3)	20.5 (16.0–25.0)
Kerman	26.3 (20.0–32.6)	17.9 (12.7–23.0)	20.9 (15.2–26.5)	22.8 (17.0–28.5)	22.0 (17.9–26.1)
Kermanshah	26.4 (17.7–35.1)	22.4 (15.5–29.3)	20.1 (10.9–29.4)	25.0 (18.5–31.6)	24.1 (18.6–29.6)
Khuzestan	29.4 (23.4–35.4)	23.9 (18.8–29.0)	24.0 (16.5–31.6)	27.2 (22.5–32.0)	26.4 (22.4–30.3)
Kohgiluyeh and Buyer Ahmad	21.9 (16.3–27.6)	18.9 (14.1–23.8)	17.1 (12.2–22.0)	21.2 (16.0–26.5)	19.9 (16.2–23.6)
Kordestan	17.3 (12.5–22.1)	18.9 (14.5–23.3)	17.1 (11.0–23.1)	17.9 (14.0–21.8)	17.6 (14.4–20.8)
Lorestan	21.7 (16.1–27.3)	17.8 (13.6–22.1)	16.3 (11.6–20.9)	21.0 (16.3–25.6)	19.2 (15.8–22.6)
Markazi	20.9 (14.1–27.7)	17.7 (12.1–23.3)	21.8 (13.3–30.4)	18.5 (13.4–23.6)	19.6 (15.1–24.0)
Mazandaran	29.2 (22.0–36.4)	27.4 (21.2–33.5)	29.5 (21.2–37.9)	28.3 (22.6–34.0)	28.5 (23.8–33.1)
North Khorasan	23.8 (18.5–29.0)	22.7 (18.1–27.4)	20.0 (14.9–25.0)	25.0 (20.3–29.8)	23.1 (19.6–26.6)
Qazvin	21.5 (14.9–28.0)	22.5 (16.9–28.0)	27.7 (19.1–36.2)	20.0 (15.2–24.7)	22.1 (17.8–26.3)
Qom	19.6 (13.9–25.4)	25.7 (19.4–32.0)	19.2 (7.7–30.7)	22.8 (18.2–27.5)	22.9 (18.6–27.2)
Razavi Khorasan	31.6 (26.8–36.5)	26.7 (22.6–30.7)	25.7 (20.7–30.7)	29.5 (25.7–33.3)	28.8 (25.7–32.0)
Semnan	27.9 (20.2–35.7)	29.0 (21.8–36.2)	26.4 (17.8–35.0)	29.5 (23.1–35.9)	28.7 (23.4–34.0)
Sistan and Baluchestan	28.1 (21.4–34.7)	20.2 (15.2–25.2)	18.6 (14.0–23.2)	29.4 (22.3–36.5)	23.5 (19.4–27.7)
South Khorasan	20.2 (14.3–26.1)	16.7 (13.0–20.4)	13.3 (8.6–18.0)	20.7 (16.2–25.2)	17.6 (14.4–20.8)
Tehran	26.0 (20.1–31.9)	21.9 (18.0–25.8)	26.4 (15.9–37.0)	23.0 (19.3–26.6)	23.2 (19.8–26.7)
West Azarbaijan	20.0 (14.5–25.4)	19.2 (14.6–23.9)	15.2 (10.5–19.8)	21.6 (16.7–26.4)	19.5 (16.0–23.1)
Yazd	14.9 (9.5–20.3)	19.3 (13.8–24.9)	20.5 (10.4–30.5)	16.9 (12.3–21.5)	17.1 (13.1–21.1)
Zanjan	17.1 (13.0–21.2)	15.5 (12.3–18.8)	14.5 (10.1–18.9)	17.0 (13.7–20.3)	16.1 (13.5–18.6)
**National**	26.4 (24.9–27.8)	23.5 (22.3–24.8)	22.9 (21.5–24.2)	25.4 (24.3–26.6)	24.8 (23.9–25.7)

Supplementary table 5. Prevalence of diabetes awareness among all with diabetes in Iran STEPS Survey 2021.

**Diabetes awareness**		**Prevalence% (95% confidence interval)**		
**Categories**	**Subcategories**	**Male**	**Female**	**Both**
Age	25 - 34	30.5 (10.6–50.3)	46.4 (27.6–65.3)	38.8 (24.4–53.2)
	35 - 44	64.8 (53.1–76.4)	64.3 (51–77.6.0)	64.5 (55.3–73.6)
	45 - 54	66.2 (57.3–75.0)	72.5 (65.7–79.4)	70.0 (64.6–75.4)
	55 - 64	72.6 (65.9–79.3)	82.4 (77.8–86.9)	78.4 (74.6–82.2)
	65 - 74	77.9 (70.9–84.9)	79.7 (74.0–85.3)	79.0 (74.5–83.4)
	≥75	63.0 (48.9–77.2)	80.4 (70.0–90.8)	70.2 (60.7–79.7)
Residency	Rural	65.7 (57.9–73.4)	73.5 (68.0–78.9)	70.6 (66.1–75.1)
	Urban	69.6 (65.1–74.2)	77.2 (73.6–80.9)	73.9 (71.1–76.8)
Wealth Index (quintiles)	First (Poorest)	64.1 (53.9–74.3)	76.4 (71.1–81.6)	72.5 (67.7–77.4)
	Second	61.8 (52.2–71.4)	78.3 (71.8–84.9)	72.0 (66.4–77.6)
	Third	75.3 (67.8–82.8)	75.6 (69.1–82.2)	75.5 (70.5–80.4)
	Fourth	60.7 (51.7–69.8)	77.7 (69.0–86.4)	69.9 (63.6–76.1)
	Fifth (Wealthiest)	79.1 (72.2–85.9)	74.4 (65.3–83.4)	77.0 (71.4–82.6)
Education (years of schooling)	0	57.0 (46.9–67.2)	79.5 (75.3–83.8)	74.6 (70.5–78.8)
	1-6	72.2 (65.4–79.0)	82.0 (77.6–86.4)	78.4 (74.6–82.1)
	7-11	74.9 (66.8–83.0)	66.9 (57.8–76.0)	70.8 (64.6–77.0)
	≥12	67.4 (60.3–74.4)	67.3 (57.6–77.0)	67.4 (61.6–73.1)
Basic insurance	No	65.6 (48.4–82.9)	50.5 (32.7–68.2)	57.4 (44.1–70.6)
	Yes	69.2 (65.1–73.3)	77.9 (75.0–80.9)	74.3 (71.8–76.7)
Complementary insurance	No	63.2 (57.8–68.7)	70.4 (66.1–74.8)	67.4 (64.0–70.8)
	Yes	76.6 (70.9–82.3)	84.2 (80.0–88.3)	81.0 (77.6–84.4)
**Total**		69.0 (65.0–73.0)	76.4 (73.3–79.5)	73.3 (70.8–75.7)

Supplementary table 6. Prevalence of diabetes treatment coverage among all with diabetes in Iran STEPS Survey 2021.

**Diabetes treatment coverage**		**Prevalence% (95% confidence interval)**		
**Categories**	**Subcategories**	**Male**	**Female**	**Both**
Age	25 - 34	18.9 (2.8–35.0)	30.6 (13.7–47.5)	25.0 (13.0–37.0)
	35 - 44	55.2 (42.3–68.1)	52.6 (39.2–65.9)	53.7 (44.2–63.2)
	45 - 54	59.5 (50.5–68.6)	64.2 (57.3–71.1)	62.3 (56.8–67.8)
	55 - 64	61.2 (53.2–69.2)	76.8 (72.0–81.6)	70.4 (66.0–74.8)
	65 - 74	70.8 (62.7–79.0)	70.5 (63.8–77.2)	70.6 (65.5–75.8)
	≥75	58.6 (44.2–73.0)	73.5 (61.9–85.0)	64.7 (55.0–74.5)
Residency	Rural	56.2 (48.0–64.3)	66.6 (60.9–72.3)	62.8 (58.1–67.5)
	Urban	61.3 (56.3–66.2)	68.9 (65.0–72.8)	65.6 (62.5–68.7)
Wealth Index (quintiles)	First (Poorest)	54.1 (43.5–64.8)	68.1 (62.4–73.9)	63.8 (58.6–69.0)
	Second	54.4 (44.7–64.0)	69.9 (62.8–76.9)	63.9 (58.0–69.8)
	Third	66.5 (58.4–74.6)	66.2 (58.7–73.8)	66.4 (60.8–71.9)
	Fourth	51.3 (42.3–60.4)	71.3 (62.6–80.0)	62.1 (55.8–68.4)
	Fifth (Wealthiest)	71.3 (62.1–80.4)	69.1 (59.9–78.4)	70.3 (63.8–76.9)
Education (years of schooling)	0	52.1 (42.0–62.1)	71.1 (66.0–76.1)	66.9 (62.3–71.5)
	1-6	63.7 (56.6–70.8)	73.6 (68.8–78.3)	69.9 (65.9–73.9)
	7-11	65.4 (56.0–74.8)	60.9 (51.6–70.2)	63.1 (56.4–69.8)
	≥12	58.3 (50.5–66.1)	59.0 (49.2–68.8)	58.6 (52.5–64.7)
Basic insurance	No	55.0 (36.9–73.2)	45.2 (28.6–61.8)	49.7 (36.9–62.4)
	Yes	60.9 (56.4–65.4)	69.7 (66.5–72.9)	66.0 (63.3–68.6)
Complementary insurance	No	53.4 (47.7–59.2)	61.3 (56.8–65.8)	58.0 (54.5–61.5)
	Yes	69.8 (63.0–76.5)	77.5 (73.0–82.0)	74.3 (70.4–78.1)
**Total**		60.4 (56.1–64.8)	68.4 (65.1–71.7)	65.0 (62.4–67.7)

Supplementary table 7. Prevalence of good (HbA1C<7%) and fair (HbA1C<8%) glycemic control among all with diabetes in Iran STEPS Survey 2021.

Glycemic control		Prevalence% (95% confidence interval)					
**Criteria**		**HbA1C<7%**			**HbA1C<8%**		
**Categories**	**Sub categories**	**Male**	**Female**	**Both**	**Male**	**Female**	**Both**
Age	25 - 34	25.1 (0.0–61.4)	61.8 (30.6–93.1)	48.6 (22.2–75.0)	49.4 (5.0–93.8)	68.2 (38.0–98.4)	61.4 (35.8–87.0)
	35 - 44	27.9 (12.5–43.2)	30.6 (16.7–44.4)	29.4 (19.1–39.7)	37.3 (19.2–55.5)	45.6 (28.2–63.0)	42.1 (29.5–54.8)
	45 - 54	28.7 (17.2–40.2)	26.5 (19.0–34.1)	27.4 (21.0–33.7)	45.8 (33.2–58.4)	53.3 (44.9–61.7)	50.4 (43.3–57.6)
	55 - 64	25.0 (17.2–32.7)	29.0 (22.8–35.2)	27.6 (22.7–32.5)	52.0 (40.8–63.2)	51.9 (45.4–58.4)	51.9 (46.1–57.7)
	65 - 74	26.4 (17.0–35.8)	28.9 (21.0–36.7)	27.9 (21.8–33.9)	49.0 (38.2–59.8)	54.5 (46.1–62.8)	52.3 (45.6–58.9)
	≥75	24.6 (3.8–45.4)	30.7 (15.5–45.9)	27.5 (14.4–40.5)	61.6 (43.6–79.6)	52.8 (36.0–69.6)	57.5 (44.8–70.2)
Residency	Rural	30.4 (18.6–42.1)	23.3 (17.5–29.1)	25.5 (20.0–31.0)	44.3 (32.5–56.0)	47.2 (40.0–54.4)	46.3 (40.1–52.4)
	Urban	25.7 (20.2–31.2)	30.5 (26.0–35.0)	28.5 (25.0–32.0)	50.4 (43.5–57.3)	53.9 (49.1–58.8)	52.5 (48.5–56.5)
Wealth Index (quintiles)	First (Poorest)	23.9 (12.8–35.0)	30.7 (23.3–38.1)	28.9 (22.6–35.2)	52.6 (36.9–68.4)	48.3 (40.4–56.2)	49.4 (42.2–56.6)
	Second	30.0 (18.8–41.3)	27.8 (19.3–36.4)	28.5 (21.7–35.4)	55.5 (42.9–68.1)	51.2 (41.5–60.9)	52.5 (44.7–60.3)
	Third	21.0 (12.4–29.5)	21.4 (14.7–28.0)	21.2 (15.9–26.5)	40.9 (30.2–51.6)	43.1 (34.5–51.7)	42.1 (35.3–48.8)
	Fourth	24.0 (13.2–34.8)	29.9 (20.9–38.9)	27.7 (20.7–34.6)	45.4 (33.5–57.4)	52.2 (42.9–61.4)	49.6 (42.3–57.0)
	Fifth (Wealthiest)	29.9 (18.7–41.1)	34.0 (22.6–45.5)	31.7 (23.5–39.8)	53.4 (39.6–67.2)	66.1 (55.0–77.2)	58.8 (49.4–68.3)
Education (years of schooling)	0	21.7 (11.5–31.8)	25.4 (20.1–30.7)	24.8 (20.1–29.5)	48.4 (35.5–61.3)	47.5 (41.4–53.6)	47.6 (42.1–53.2)
	1-6	21.0 (13.9–28.0)	30.7 (24.6–36.8)	27.5 (22.7–32.2)	42.8 (33.5–52.0)	52.1 (45.6–58.7)	49.0 (43.6–54.4)
	7-11	27.6 (14.7–40.4)	28.4 (15.9–41.0)	28.0 (19.0–37.0)	52.1 (37.2–67.0)	51.2 (38.5–63.9)	51.7 (41.8–61.5)
	≥12	31.7 (22.3–41.0)	29.8 (19.6–40.0)	30.9 (24.0–37.8)	53.6 (42.6–64.6)	61.2 (48.9–73.5)	56.7 (48.4–64.9)
Basic insurance	No	20.5 (3.8–37.2)	20.1 (6.6–33.5)	20.3 (9.7–30.9)	59.7 (34.2–85.2)	32.9 (16.9–48.9)	45.7 (29.1–62.4)
	Yes	26.9 (21.7–32.2)	29.0 (25.2–32.8)	28.2 (25.1–31.3)	48.8 (42.5–55.0)	53.0 (48.7–57.2)	51.4 (47.8–54.9)
Complement ary insurance	No	34.1 (26.1–42.1)	28.3 (23.6–33.1)	30.6 (26.3–34.8)	52.0 (43.5–60.6)	51.0 (45.6–56.4)	51.4 (46.7–56.1)
	Yes	19.1 (13.5–24.7)	29.0 (23.2–34.9)	25.1 (20.9–29.4)	47.2 (38.4–56.0)	53.2 (46.8–59.6)	50.8 (45.6–56.0)
**Total**		26.4 (21.4–31.4)	29.0 (25.2–32.8)	28.0 (25.0–31.0)	49.5 (43.4–55.6)	52.5 (48.4–56.7)	51.4 (47.9–54.8)

Supplementary table 8. Estimated number of the population with diabetes and prediabetes based on Iran STEPS Survey 2021.

**Burden**		**Number (95% confidence interval)**	
**Categories**	**Sub categories**	**Diabetes**	**Prediabetes**
Age	25 - 34	357450 (250554–464346)	2670463 (2380509–2960418)
	35 - 44	712436 (580630–844243)	2890064 (2662301–3117827)
	45 - 54	1395890 (1253960–1537821)	2424112 (2255834–2592391)
	55 - 64	1572922 (1445722–1700123)	1685376 (1555663–1815089)
	65 - 74	772001 (695226–848775)	857449 (768833–946064)
	≥75	487786 (394969–580603)	657360 (531675–783046)
Sex	Women	3583175 (3352292–3814058)	5733017 (5435069–6030966)
	Men	3315849 (3039063–3592636)	6497754 (6144494–6851014)
Residency	Rural	1316830 (1197766–1435893)	2744648 (2585049–2904247)
	Urban	5622742 (5287417–5958067)	9405730 (8977856–9833603)
Wealth Index (quintiles)	First (Poorest)	1304606 (1167419–1441793)	2585106 (2360464–2809748)
	Second	1517931 (1347154–1688708)	2360107 (2161225–2558989)
	Third	1321583 (1181493–1461674)	2443625 (2254750–2632500)
	Fourth	1426314 (1263924–1588705)	2551255 (2352611–2749899)
	Fifth (Wealthiest)	1381417 (1189532–1573301)	2232883 (2002476–2463290)
**Total**		6935886 (6580648–7291123)	12148229 (11692071–12604387)

Supplementary table 9. Health-related quality of life assessment in all diabetic patients included in Iran STEPS Survey 2021.

Quality of life domain	Prevalence% (95% confidence interval)		
	No problem	Slightly affected	Debilitated
Mobility	84.7 (82.9–86.5)	14.9 (13.1–16.7)	0.4 (0.2–0.7)
Self-care	94.7 (93.7–95.6)	4.7 (3.8–5.6)	0.7 (0.3–1.0)
Usual activities	89.7 (88.3–91.1)	9.3 (7.9–10.6)	1.0 (0.6–1.5)
Pain/discomfort	43.4 (40.6–46.2)	46.5 (43.8–49.3)	10.1 (8.6–11.6)
Anxiety/depression	55.5 (52.7–58.3)	35.7 (33.0–38.5)	8.8 (7.4–10.1)

Supplementary table 10. Prevalence of owning a glucometer by patients with diabetes included in Iran STEPS Survey 2021.

**Glucometer**		**Prevalence% (95% confidence interval)**		
**Categories**	**Sub categories**	**Male**	**Female**	**Both**
Age	25 - 34	30.7 (10.4–51.0)	15.5 (2.4–28.5)	22.8 (11.0–34.5)
	35 - 44	32.0 (20.8–43.2)	38.2 (24.6–51.7)	35.6 (26.3–44.8)
	45 - 54	50.3 (40.9–59.6)	46.5 (39.5–53.5)	48.0 (42.4–53.6)
	55 - 64	49.3 (40.9–57.6)	55.6 (49.9–61.2)	53.0 (48.2–57.8)
	65 - 74	51.0 (42.0–59.9)	50.1 (43.0–57.2)	50.4 (44.9–56.0)
	≥75	42.7 (27.9–57.5)	40.1 (25.8–54.5)	41.6 (31.2–52.1)
Residency	Rural	36.1 (28.0–44.3)	34.5 (28.8–40.2)	35.1 (30.4–39.8)
	Urban	48.9 (43.8–54.1)	52.2 (48.1–56.3)	50.8 (47.5–54.0)
Wealth Index (quintiles)	First (Poorest)	30.7 (21.3–40.0)	32.7 (26.6–38.8)	32.1 (26.9–37.2)
	Second	40.8 (31.1–50.5)	51.5 (43.7–59.4)	47.4 (41.2–53.6)
	Third	52.1 (43.5–60.7)	47.2 (39.7–54.8)	49.4 (43.7–55.1)
	Fourth	43.6 (34.7–52.4)	59.0 (50.2–67.7)	51.9 (45.6–58.1)
	Fifth (Wealthiest)	58.0 (46.8–69.1)	58.4 (48.4–68.5)	58.2 (50.5–65.8)
Education (years of schooling)	0	33.7 (24.7–42.8)	40.0 (34.7–45.3)	38.6 (34.0–43.2)
	1-6	48.9 (41.6–56.3)	51.0 (45.5–56.5)	50.2 (45.8–54.6)
	7-11	49.3 (38.4–60.2)	45.4 (35.8–55.1)	47.3 (40.1–54.6)
	≥12	48.0 (40.0–56.0)	57.6 (47.9–67.3)	51.8 (45.6–58.1)
Basic insurance	No	32.5 (16.4–48.7)	31.8 (18.1–45.6)	32.2 (21.7–42.7)
	Yes	47.9 (43.2–52.6)	49.3 (45.7–52.8)	48.7 (45.9–51.5)
Complementary insurance	No	42.7 (36.8–48.6)	36.4 (32.2–40.7)	39.1 (35.6–42.6)
	Yes	52.4 (45.0–59.9)	64.1 (58.9–69.2)	59.2 (54.7–63.7)
**Total**		46.8 (42.3–51.3)	48.5 (45.0–51.9)	47.8 (45.0–50.5)

Supplementary table 11. Mean onset age of diabetes mellitus among patients included in Iran STEPS Survey 2021.

**Onset age**		**Mean (95% confidence interval)**		
**Categories**	**Sub categories**	**Male**	**Female**	**Both**
Age	25 - 34	27.7 (21.2–34.2)	28.4 (25.8–30.9)	28.1 (25.2–31.0)
	35 - 44	35.3 (34.0–36.6)	33.4 (29.4–37.4)	34.2 (31.7–36.7)
	45 - 54	42.3 (40.4–44.2)	40.9 (39.6–42.1)	41.4 (40.3–42.5)
	55 - 64	49.3 (47.7–50.9)	46.8 (45.1–48.5)	47.7 (46.5–49.0)
	65 - 74	55.5 (53.7–57.3)	53.1 (51.3–54.9)	54.1 (52.7–55.4)
	≥75	63.3 (56.9–69.8)	63.3 (60.3–66.4)	63.3 (59.6–67.0)
Residency	Rural	51.2 (48.5–53.9)	47.5 (45.9–49.1)	48.8 (47.4–50.2)
	Urban	48.4 (47.0–49.8)	46.0 (44.6–47.3)	47.0 (46.0–48.0)
Wealth Index (quintiles)	First (Poorest)	49.4 (46.0–52.8)	47.8 (45.8–49.9)	48.3 (46.5–50.0)
	Second	51.0 (48.3–53.7)	46.7 (43.6–49.8)	48.1 (45.8–50.5)
	Third	49.4 (46.7–52.2)	44.6 (42.5–46.7)	46.8 (45.1–48.5)
	Fourth	48.0 (46.0–50.1)	45.9 (43.4–48.4)	46.7 (45.0–48.5)
	Fifth (Wealthiest)	47.8 (45.0–50.5)	45.3 (42.1–48.5)	46.7 (44.7–48.8)
Education (years of schooling)	0	61.5 (57.9–65.1)	51.2 (49.7–52.7)	52.9 (51.5–54.4)
	1-6	50.6 (48.8–52.4)	46.1 (44.5–47.7)	47.7 (46.4–48.9)
	7-11	47.0 (43.9–50.1)	41.6 (39.4–43.9)	44.4 (42.4–46.4)
	≥12	45.3 (43.5–47.2)	40.5 (36.8–44.2)	43.4 (41.5–45.3)
Basic insurance	No	46.2 (42.4–49.9)	45.2 (40.7–49.7)	45.7 (42.8–48.6)
	Yes	49.0 (47.7–50.4)	46.2 (45.1–47.4)	47.3 (46.4–48.2)
Complementary insurance	No	48.5 (46.8–50.2)	46.0 (44.7–47.2)	47.0 (46.0–48.0)
	Yes	49.3 (47.4–51.3)	46.5 (44.5–48.5)	47.6 (46.2–49.0)
**Total**		48.8 (47.6–50.1)	46.3 (45.1–47.4)	47.3 (46.4–48.1)

Supplementary table 12. Prevalence of hypoglycemic event in past two weeks in patients with diabetes included in Iran STEPS Survey 2021.

**Hypoglycemic event**		**Prevalence% (95% confidence interval)**		
**Categories**	**Sub categories**	**Male**	**Female**	**Both**
Age	25 - 34	11.7 (0.0–29.8)	33.6 (4.0–63.3)	25.7 (4.8–46.7)
	35 - 44	28.0 (2.9–53.1)	14.6 (5.7–23.6)	20.4 (7.4–33.5)
	45 - 54	14.7 (6.5–23.0)	21.7 (15.3–28.2)	19.1 (13.9–24.2)
	55 - 64	13.9 (7.5–20.3)	21.3 (16.1–26.5)	18.7 (14.5–22.8)
	65 - 74	18.5 (10.4–26.6)	18.8 (11.7–25.9)	18.7 (13.3–24.1)
	≥75	25.7 (4.6–46.9)	28.7 (12.2–45.2)	27.1 (13.5–40.7)
Residency	Rural	22.3 (11.3–33.3)	22.7 (16.8–28.6)	22.6 (17.2–27.9)
	Urban	16.7 (11.4–22.1)	20.4 (16.6–24.3)	18.9 (15.8–22.1)
Wealth Index (quintiles)	First (Poorest)	28.0 (9.9–46.1)	24.2 (17.3–31.1)	25.2 (18.2–32.3)
	Second	16.8 (7.3–26.2)	25.6 (17.9–33.3)	22.7 (16.7–28.7)
	Third	23.4 (14.4–32.4)	24.3 (16.3–32.2)	23.9 (17.9–29.8)
	Fourth	14.6 (6.1–23.1)	13.4 (7.8–18.9)	13.8 (9.1–18.6)
	Fifth (Wealthiest)	12.7 (2.9–22.6)	17.8 (7.7–27.9)	14.9 (7.8–22.0)
Education (years of schooling)	0	15.7 (5.8–25.6)	19.1 (14.4–23.8)	18.5 (14.3–22.8)
	1-6	17.7 (11.6–23.9)	27.0 (21.0–33.1)	23.9 (19.3–28.4)
	7-11	20.6 (7.1–34.0)	13.8 (6.9–20.7)	17.2 (9.4–25.0)
	≥12	16.2 (7.4–25.0)	15.6 (7.5–23.7)	16.0 (9.8–22.2)
Basic insurance	No	46.5 (19.5–73.5)	18.6 (5.4–31.7)	32.7 (15.3–50.1)
	Yes	15.6 (11.0–20.1)	21.1 (17.7–24.5)	19.0 (16.2–21.7)
Complementary insurance	No	17.1 (10.5–23.7)	18.9 (14.9–23.0)	18.2 (14.7–21.8)
	Yes	17.3 (10.1–24.4)	23.3 (18.0–28.6)	20.9 (16.6–25.2)
**Total**		17.6 (12.7–22.4)	20.9 (17.6–24.2)	19.6 (16.9–22.4)

Supplementary table 13. Prevalence of positive family history of diabetes among all participants in Iran STEPS Survey 2021.

**Positive family history**		**Prevalence% (95% confidence interval)**		
**Categories**	**Sub categories**	**Male**	**Female**	**Both**
Age	25 - 34	26.7 (24.7–28.8)	29.2 (27.3–31.1)	28.1 (26.8–29.5)
	35 - 44	30.8 (28.8–32.8)	36.1 (34.3–37.8)	33.8 (32.5–35.2)
	45 - 54	30.2 (28.1–32.4)	36.5 (34.6–38.4)	33.8 (32.4–35.3)
	55 - 64	29.6 (27.3–31.8)	35.8 (33.7–37.9)	33.0 (31.4–34.5)
	65 - 74	27.8 (24.9–30.7)	30.9 (28.2–33.6)	29.5 (27.5–31.4)
	≥75	23.8 (19.9–27.7)	29.7 (25.0–34.4)	26.4 (23.4–29.4)
Residency	Rural	25.8 (24.0–27.6)	32.0 (30.3–33.7)	29.3 (28.0–30.5)
	Urban	29.9 (28.8–31.1)	34.6 (33.6–35.6)	32.5 (31.8–33.3)
Wealth Index (quintiles)	First (Poorest)	23.1 (21.0–25.2)	28.4 (26.5–30.3)	26.2 (24.8–27.6)
	Second	30.4 (28.2–32.7)	35.0 (33.0–37.0)	33.1 (31.6–34.6)
	Third	29.2 (27.1–31.4)	33.9 (31.7–36.0)	31.6 (30.1–33.1)
	Fourth	30.2 (28.1–32.4)	37.8 (35.6–40.0)	34.2 (32.6–35.7)
	Fifth (Wealthiest)	30.7 (28.6–32.8)	34.7 (32.6–36.8)	32.8 (31.3–34.3)
Education (years of schooling)	0	21.6 (18.8–24.4)	28.4 (26.5–30.4)	26.5 (24.9–28.1)
	1-6	26.9 (25.0–28.8)	35.1 (33.4–36.8)	31.8 (30.5–33.0)
	7-11	31.4 (29.3–33.4)	39.4 (37.1–41.7)	35.3 (33.7–36.8)
	≥12	30.3 (28.9–31.8)	33.6 (32.1–35.1)	32.0 (31.0–33.0)
Basic insurance	No	27.9 (25.0–30.8)	31.0 (28.1–34.0)	29.5 (27.4–31.5)
	Yes	29.1 (28.1–30.1)	34.3 (33.3–35.2)	32.0 (31.3–32.7)
Complementary insurance	No	28.0 (26.8–29.1)	31.9 (30.9–32.9)	30.1 (29.4–30.9)
	Yes	31.2 (29.4–33.1)	38.8 (37.1–40.5)	35.5 (34.3–36.8)
**Total**		28.9 (28.0–29.9)	34.0 (33.1–34.9)	31.7 (31.1–32.4)

Supplementary table 14. Prevalence of positive family history of diabetes mellitus among all participants with diabetes included in Iran STEPS Survey 2021.

**Positive family history**		**Prevalence% (95% confidence interval)**		
**Categories**	**Sub categories**	**Male**	**Female**	**Both**
Age	25 - 34	52.2 (28.2–76.3)	60.3 (42.6–78.1)	56.4 (41.5–71.3)
	35 - 44	75.3 (65.7–85.0)	67.2 (53.8–80.6)	70.6 (61.6–79.6)
	45 - 54	61.4 (52.3–70.4)	57.8 (50.7–64.8)	59.2 (53.6–64.8)
	55 - 64	47.8 (39.4–56.2)	59.3 (53.7–64.9)	54.7 (49.7–59.6)
	65 - 74	50.5 (41.5–59.5)	51.3 (44.1–58.6)	51.0 (45.4–56.7)
	≥75	45.1 (29.6–60.7)	58.3 (44.3–72.2)	50.6 (39.7–61.5)
Residency	Rural	53.3 (45.2–61.5)	52.4 (46.4–58.5)	52.7 (47.9–57.6)
	Urban	54.5 (49.1–59.8)	59.5 (55.4–63.6)	57.3 (54.0–60.6)
Wealth Index (quintiles)	First (Poorest)	45.6 (34.7–56.5)	49.7 (43.2–56.2)	48.4 (42.7–54.0)
	Second	54.6 (44.7–64.5)	60.7 (53.1–68.2)	58.4 (52.4–64.5)
	Third	53.8 (44.8–62.8)	62.5 (55.3–69.7)	58.7 (52.9–64.4)
	Fourth	56.1 (47.2–65.1)	55.9 (47.0–64.8)	56.0 (49.7–62.3)
	Fifth (Wealthiest)	57.3 (46.0–68.6)	60.1 (50.4–69.8)	58.5 (50.9–66.2)
Education (years of schooling)	0	38.4 (28.5–48.3)	50.6 (45.2–55.9)	48.0 (43.2–52.8)
	1-6	51.4 (43.9–58.9)	60.0 (54.5–65.4)	56.8 (52.3–61.2)
	7-11	61.5 (50.7–72.2)	68.8 (60.0–77.6)	65.2 (58.3–72.1)
	≥12	57.3 (48.9–65.7)	58.9 (49.1–68.7)	58.0 (51.6–64.4)
Basic insurance	No	37.6 (20.3–54.9)	33.6 (19.3–48.0)	35.4 (24.3–46.6)
	Yes	55.3 (50.5–60.1)	59.7 (56.2–63.1)	57.9 (55.0–60.7)
Complementary insurance	No	54.3 (48.3–60.2)	54.1 (49.5–58.6)	54.2 (50.5–57.8)
	Yes	54.5 (47.0–62.0)	63.4 (58.3–68.6)	59.7 (55.3–64.1)
**Total**		54.3 (49.6–58.9)	58.0 (54.5–61.5)	56.4 (53.6–59.3)

Supplementary figure 1. Subnational prevalence of (A) diabetes, and (B) prediabetes among provinces in Iran STEPS Survey 2021.

A

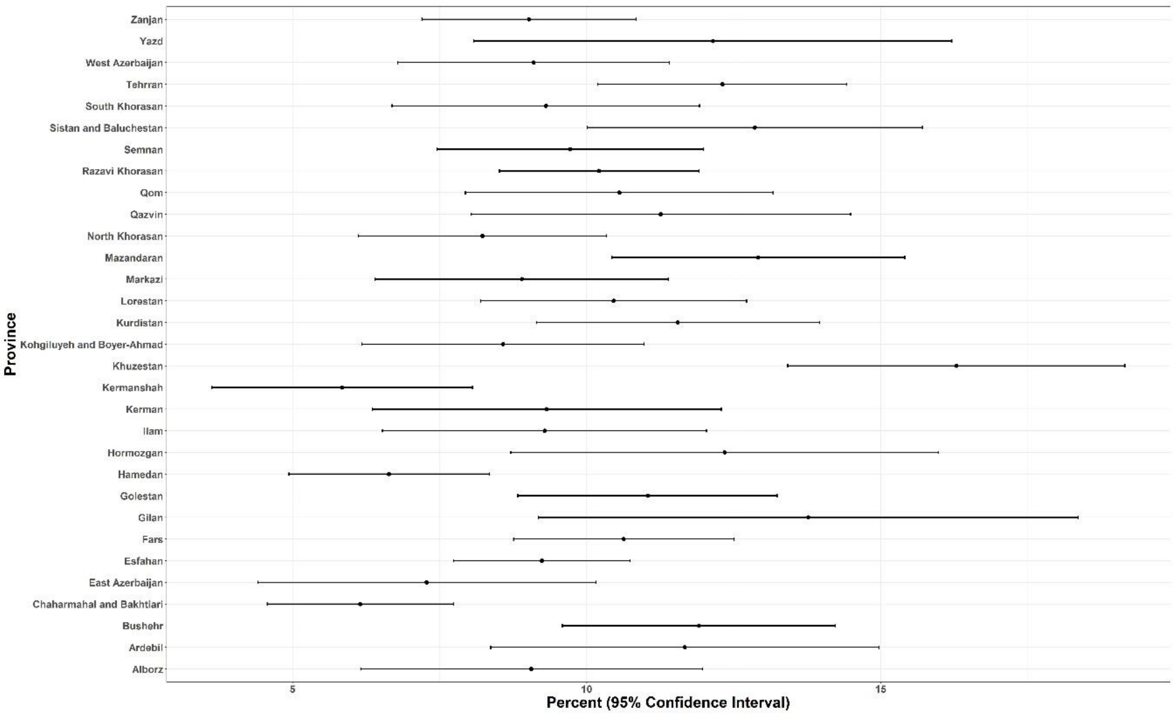

B

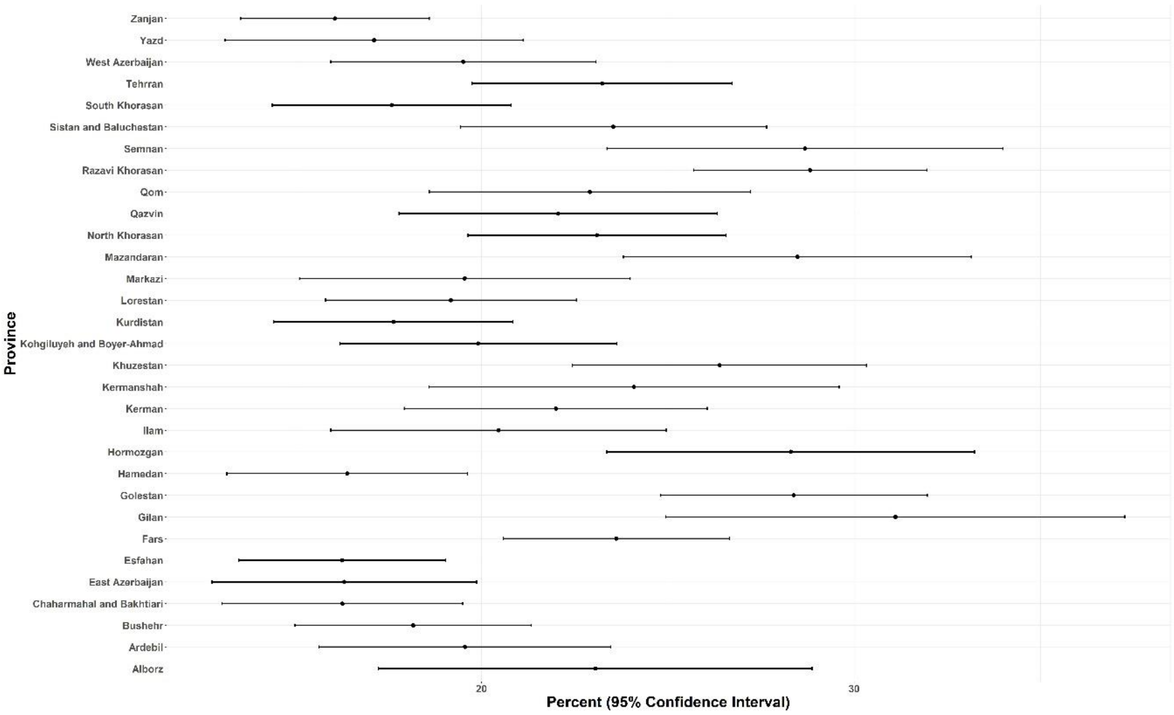

Supplementary figure 2. Prevalence of (A) diabetes, and (B) prediabetes in different age categories of each sex in Iran STEPS Survey 2021.

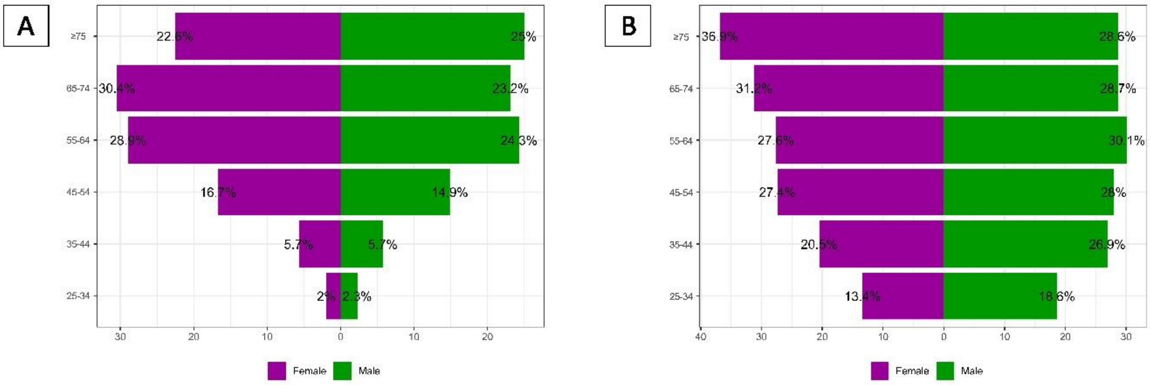

Supplement: Supplementary file 1 — Supplementary Information. [file 41598_2023_39433_MOESM1_ESM.pdf]
